# Electrochemical Anodization for the Fabrication of Wafer‐Scale p‐Type Organic Permeable Base Transistors Arrays with MHz Operation

**DOI:** 10.1002/adma.202419974

**Published:** 2025-05-06

**Authors:** Juan Wang, Amric Bonil, Jan Frede, Lautaro Petrauskas, Jörn Vahland, Tobias Antrack, Christian Matthus, Wooik Jang, Hans Kleemann

**Affiliations:** ^1^ Dresden Integrated Center for Applied Physics and Photonic Materials (IAPP) Technische Universität Dresden Nöthnitzer Straße 61 01187 Dresden Germany; ^2^ Chair for Circuit Design and Network Theory (CCN), Faculty of Electrical and Computer Engineering Technische Universität Dresden 01069 Dresden Germany; ^3^ Nano and Microelectronic System (NMES) Technische Universität Ilmenau 98693 Ilmenau Germany

**Keywords:** complementary inverter, electrochemical anodization, p‐type organic permeable base transistors (OPBTs), vertical organic transistors, wafer‐scale transistor fabrication

## Abstract

Organic thin‐film transistors (OTFTs) are promising for flexible, low‐cost, and biocompatible electronics. However, conventional planar OTFTs are hindered by the large channel length limiting the transconductance and switching frequencies. Vertical OTFTs, particularly organic permeable‐base transistors (OPBTs), address these challenges with short channel lengths defined by the layer thickness. While n‐type OPBTs have advanced significantly, p‐type OPBTs face challenges such as lower transmission, higher leakage currents, and unreliable fabrication processes. This work introduces a wafer‐scale method for fabricating p‐type OPBTs using electrochemical anodization of the base electrode. The anodization process applied directly atop the organic semiconductor, preserves electrical properties while suppressing base leakage. The resulting anodized OPBTs exhibit high‐performance characteristics, including an on‐current density of 301 mAcm^−2^, low leakage current of 4.32 × 10^−9^ A, maximum transmission of 99.9999%, and a maximum current gain of 1.89 × 10^6^—a 100,000‐fold improvement over prior methods. Small signal analysis reveals a cutoff frequency of 1.49 MHz, with a voltage‐normalized cutoff frequency of 0.54 MHzV^−1^. Large‐scale arrays show 96.3% fabrication yield and excellent uniformity. Complementary inverters integrating n‐ and p‐type OPBTs exhibit superior switching, highlighting the potential of anodized OPBTs for advanced applications in displays and circuits.

## Graphical abstract

This work presents a wafer‐scale fabrication method for high‐performance p‐type organic permeable base transistors (OPBTs) using electrochemical anodization. The anodized OPBTs achieve high on‐current density, low leakage, MHz‐range operation, and exceptional current gain. With a 96.3% yield in large‐area arrays, these devices enable scalable organic electronics and advanced complementary circuits, demonstrating potential for future display and logic applications.

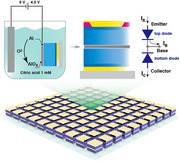

## 1 Introduction

Organic thin film transistors (OTFTs) have garnered rapid development in recent years due to their low cost, high mechanical flexibility, low processing temperature and biocompatibility, showing great application prospects in logic circuits, active matrix displays, flexible, and stretchable devices, smart sensors, radio frequency identification tags, and more.^[^ ^1^, ^2^, ^3^, ^4^, ^5^, ^6^, ^7^, ^8^, ^9^ ^]^ Despite the numerous publications and significant advancements with regard to the performance of planar OTFTs, their relatively large conducting channel lengths, typically on the micrometer scale or even larger, are a key disadvantage as it is a limiting factor for the transconductance and the maximum frequency of switching. Reducing the channel length to a smaller scale is challenging due to limitations in device geometry and processing techniques.^[^ ^10^ ^]^ One approach to solving this problem is to use vertical channel OTFTs, which redefine the channel length determination from lateral structuring to layer thickness.^[^ ^11^, ^12^, ^13^ ^]^ Current deposition methods enable precise control over layer thicknesses in the tens of nanometer range, facilitating the fabrication of transistors with very short channels.^[^ ^14^, ^15^, ^16^, ^17^, ^18^ ^]^ This eliminates the need for complex lateral structuring at the nanoscale. Consequently, vertical OTFTs can potentially achieve high current density and operational frequencies under low operating voltages, making them highly suitable for practical applications in organic electronics.^[^ ^19^, ^20^, ^21^, ^22^ ^]^

Among vertical OTFTs, organic permeable‐base transistors (OPBTs) have reached a record transition frequency of 40 MHz, exhibiting their significant potential for high‐speed switching applications.^[^ ^23^ ^]^ The OPBT shares the primary advantages of vertical OTFTs, including minimal dependence on complex lateral structuring techniques, short channel lengths, and consequently, very high on‐state current densities (*J*_*on*_) of up to 1 kAcm^−2^ for n‐type devices based on the semiconductor C60.^[^ ^24^ ^]^ Moreover, recent advancements include the demonstration of dual‐base OPBTs, which enable precise tuning of threshold voltages (*V*_*TH*_) for logic gates.^[^ ^5^ ^]^ Additionally, ring oscillators based on OPBTs have been developed, achieving a stage delay of 11 ns at a low supply voltage of 4 V.^[^ ^6^ ^]^ These achievements bring OPBTs technology remarkably close to meeting the standards required for high‐frequency wireless communication, i.e., 13.56 MHz. However, all these achievements are based on the high performance of n‐type OPBTs, which use C60 as the semiconductor material. The fabrication of p‐type devices with reliable performance has remained a significant challenge, yet they are essential for developing robust and low‐energy complementary circuits. In our previous studies, we have suggested that selecting the appropriate metallic underlayer and extending exposure to ambient air are crucial factors in promoting the formation of the grid‐like base electrode and the oxidation layer around the base in p‐type OPBTs based on pentacene as semiconductor.^[^ ^25^, ^26^ ^]^ However, due to lower transmission factor (*α* = *I*_*C*_/*I*_*E*_), current gain (*β* = *I*_*C*_/*I*_*B*_), device reproducibility, yield, uniformity, and higher base leakage current (*I*_*B*_), these devices have not yet matched the performance of n‐type OPBTs, hindering the development of efficient and fast complementary circuits. Moreover, the focus has primarily been on the performance of the best‐fabricated devices, often referred to as hero devices in previous publications on OPBTs. Hence, integrating large‐scale and high‐quality p‐type OPBTs remains a critical challenge, posing a significant technological hurdle for advancing the practical application of OPBT‐based integrated circuits.

The OPBTs, with a structure resembling a vacuum tube triode, feature a grid‐like base electrode that modulates the current flow from the emitter (*I*_*E*_) to the collector (*I*_*C*_).^[^ ^27^ ^]^ Due to mechanical strain induced during the oxidation process, nanometer‐sized pinholes open up in the base of the transistor, forming the grid‐like base electrode characteristic of the OPBTs.^[^ ^28^, ^29^ ^]^ The current injected at the emitter electrode transports through the nano pinholes in the base electrode and reaches the collector electrode. The crucial step of OPBT manufacturing is the oxidation of the base layer, which involves forming a dense oxide layer around the metal film of the base to prevent leakage currents and further enhance *α*, *J*_*on*_, and operating frequency. Previous works involved oxidizing samples in ambient air by introducing oxygen during deposition, effectively reducing current leakage to acceptable levels. Electrochemical anodization has emerged as a powerful technique for creating dense, high‐quality insulating layers on metal surfaces,^[^ ^30^, ^31^, ^32^, ^33^ ^]^ particularly on aluminum or titanium films, by controlled oxidation. This method shows great potential for improving the dielectric properties of organic transistors. While there are reports of successful anodization techniques improving the performance of lateral p‐type thin‐film transistors,^[^ ^34^, ^35^, ^36^ ^]^ these studies did not subject the semiconductor itself to the electrochemical process. Additionally, electrochemical anodization was employed on the base electrode of n‐type OPBTs to achieve enhanced and precise oxidation control.^[^ ^37^, ^38^ ^]^ This approach demonstrates that anodization is a viable method for creating an excellent and well‐defined dielectric oxide for n‐type OPBTs, exhibiting the capability of organic semiconductors to withstand this process effectively. Therefore, electrochemical anodization presents a promising way to form robust, dense oxide films and fabricate high‐performance p‐type OPBTs with reduced leakage currents. Still, there is the concern that anodization of metals atop a p‐type organic semiconductor might cause irreversible oxidation of the semiconductor, deteriorating the charge carrier transport.^[^ ^39^, ^40^ ^]^

In this work, we present a method for fabricating wafer‐scale, uniform arrays of p‐type OPBTs using an electrochemically anodized base electrode. We demonstrate that the anodization process can be directly applied to the base electrode on top of the organic semiconductor layer without damaging its electrical performance. This approach effectively suppresses the current injected at the emitter flowing into the base, significantly improving the transmission current compared to the ambient‐air oxidized base reported in our previous work.^[^ ^26^ ^]^ The anodized pentacene OPBTs, across different active areas (*A*_*act*_), exhibit reliable transistor performance, with an *J*_*on*_ of 301 mAcm^−2^, a low *I*_*B*_ of 4.32 × 10^−9^ A, a maximum transmission (*α*_*max*_) of 99.9999%, and a maximum current gain (*β*_*max*_) of 1.89 × 10^6^. This fabrication method addresses critical limitations in p‐type OPBT performance by achieving a 100‐fold reduction in the *A*_*act*_, facilitating device miniaturization. Furthermore, *I*_*B*_ was reduced by a factor of 100,000, resulting in a corresponding 100,000‐fold enhancement in *β*_*max*_. The *α*_*max*_ also improved significantly, increasing from 97% to 99.9999%, underscoring the advantages of this approach over previously reported methods.^[^ ^26^ ^]^ Impedance spectroscopy of the anodized OPBTs confirms the formation of robust oxide films around the base electrode after anodization, contributing to their reliable performance. The unity‐current gain (cutoff) frequency *f*_*T*_ was evaluated using a two‐port signal analysis under different biasing conditions. A *f*_*T*_ of 1.49 MHz was measured at base‐emitter voltages (*V*_*BE*_) and collector‐emitter voltages (*V*_*CE*_) of ‐2.75 and ‐3 V, respectively, yielding a voltage‐normalized cutoff frequency of 0.54 MHzV^−1^. In addition, large‐scale arrays of anodized pentacene OPBTs were fabricated to assess their potential for commercial applications. These devices exhibited excellent uniformity in electrical performance, with an average on‐current (*I*_*on*_) of 2.1 × 10^−4^ A, off‐current (*I*_*off*_) of 2.1 × 10^−7^ A, *I*_*B*_ of 3.1 × 10^−8^ A, and maximum transconductance (*g*_*m*, *max*_) of 0.18 mS, along with a high fabrication yield of 96.3% through simultaneous massive devices's base anodization. Furthermore, we demonstrate that anodized p‐type OPBTs can be integrated with n‐type OPBTs to realize a complementary inverter with excellent switching performance. These results highlight the strong potential of anodized OPBT devices for applications in active matrix displays and integrated circuits.

## 2 Results and Discussion

### 2.1 Electrochemical Anodization

**Figure 1:**
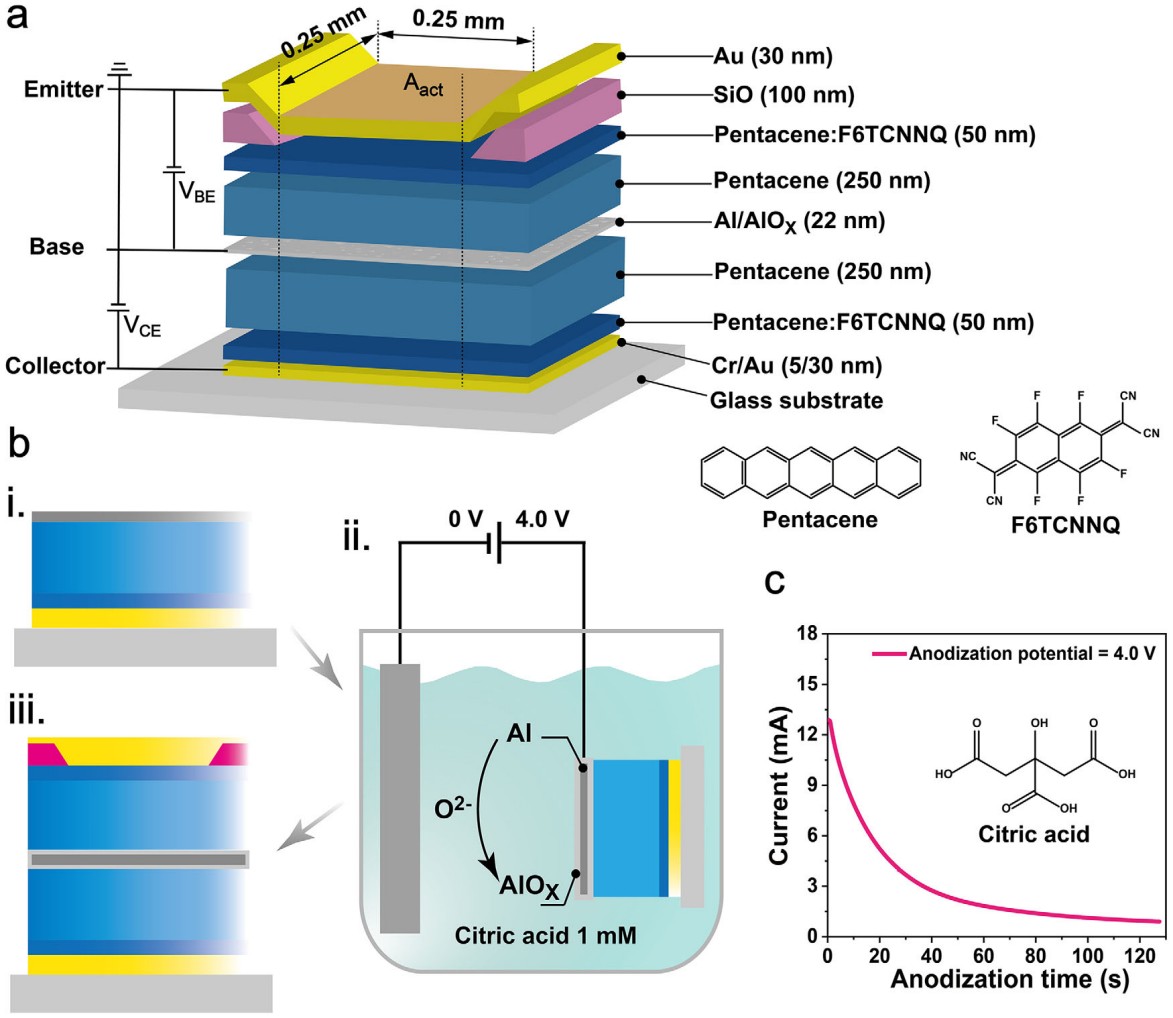
Device structure of p‐type OPBTs and fabrication process. a) Device schematics of a pentacene‐based OPBT with an electrochemically oxidized permeable base electrode. Molecular structures of the organic semiconductor pentacene and the p‐type dopant F6TCNNQ used in this study are shown in the lower right corner. b) Fabrication process of anodized pentacene OPBT: i) Thermal evaporation of the bottom half of OPBT layers through a series of shadow masks. ii) Schematic illustration of the anodization process, showing the anodizing base of the half‐fabricated OPBT and the growth of AlO_x_ on the Al surface. iii) Thermal evaporation of the remaining layers completes the transistor. c) Anodizing current between anode and counter electrode. Inset: Molecular structure of citric acid used in the anodization electrolyte solution for this study.

**Figure** **1**a shows a schematic diagram of the p‐type OPBTs with an electrochemically anodized base electrode. The transistor features three parallel electrodes–emitter, base, and collector–separated by an organic semiconductor layer. Pentacene is used as the hole‐conductive material, with F6TCNNQ‐doped pentacene inserted between the intrinsic pentacene and the metal electrode to enable an ohmic‐like contact. A layer of SiO underneath the emitter electrode is used to define the *A*_*act*_ to 0.0625 mm^2^ (0.25 mm × 0.25 mm) and 0.36 mm^2^ (0.6 mm × 0.6 mm), respectively. The thin base electrode in the middle is grown on a network‐like structured pentacene, facilitating the formation of pinholes on the base^[^ ^25^, ^26^ ^]^ (further fabrication details are given in the materials section). The bias applied to the base electrode relative to the grounded emitter modulates the hole current, which passes from the emitter electrode through the pinholes in the base to the collector electrode. The base electrode layer is typically made of an evaporated aluminum film, which develops an oxide layer after being exposed to ambient air for 15 min in the dark. However, this unreliable process results in a poor‐quality oxidation layer on the base electrode, leading to high base leakage current, low gain, and poor transmission. Although extending the exposure time of the base to ambient air is realized to improve the performance of p‐type OPBTs, prolonged exposure leads to the degradation of the p‐type material, which is undesirable.^[^ ^26^ ^]^

Electrochemical anodization of the aluminum base first proposed for application in C60‐based n‐type OPBTs, has been shown to achieve high‐performance OPBTs with significantly reduced *I*_*B*_.^[^ ^37^ ^]^ The main challenge in applying this method to p‐type OPBTs is whether the organic semiconductor can withstand the electrochemical anodization process. To explore the potential for industrial application of anodized OPBT, the 6 × 6 device arrays on a wafer scale 15 cm × 15 cm glass substrate was fabricated, allowing for anodizing of the base for many devices simultaneously. There are 34 samples in the arrays, each containing four pixels, divided into two groups that share a common anodization pad. As shown in Figure 1b and Figure S1 (Supporting Information), the anodization process involved using the base‐covered anodization pads of half of the arrays as anodes, with an aluminum sheet slightly larger than half of the substrate serving as the counter electrode. During the anodization process, half‐fabricated samples (collector electrode, bottom doped pentacene layer, bottom intrinsic pentacene, and base electrode) are immersed in a 1 mM citric acid solution with the anode at 4 V and the counter electrode grounded. A uniform electric field can be created between the anode and counter electrode, ensuring an identical thickness of the oxidation layer around the base electrode in these simultaneously processed OPBTs. After applying the anodization potential, an AlO_x_ layer forms at the interface between the base electrode and the acid solution until it reaches saturation. This occurs because, over time, the growing AlO_x_ layer surrounds the base, blocking the anodization reaction between the aluminum base and the acid solution, which can be observed as the anodization current decreases to a plateau (Figure 1c). Details on device fabrication and anodization process are provided in the experiment section. It is worth pointing out, that the half‐fabricated devices were preheated at 120 °C for 2 min to improve adhesion between the pentacene and the base electrode while keeping the annealing time short to avoid degradation of pentacene under high temperatures.^[^ ^41^ ^]^ This process to improve the adhesion is necessary to avoid delamination of the organic layer from the substrate under anodization conditions. For the conditions defined here, no delamination is observed, but it happens frequently for samples annealed at lower temperatures. To further improve the adhesion, a nail protection polymeric coating can be employed (see materials sections). To investigate whether the anodization process affects the thin film morphology, we conducted Scanning Electron Microscopy (SEM) analysis. As shown in Figure S2 (Supporting Information), the SEM images confirm that the morphology of the thin films remains unchanged after anodization, showing no noticeable difference compared to non‐anodized devices.^[^ ^25^, ^26^ ^]^

### 2.2 Electrical Performance

**Figure 2:**
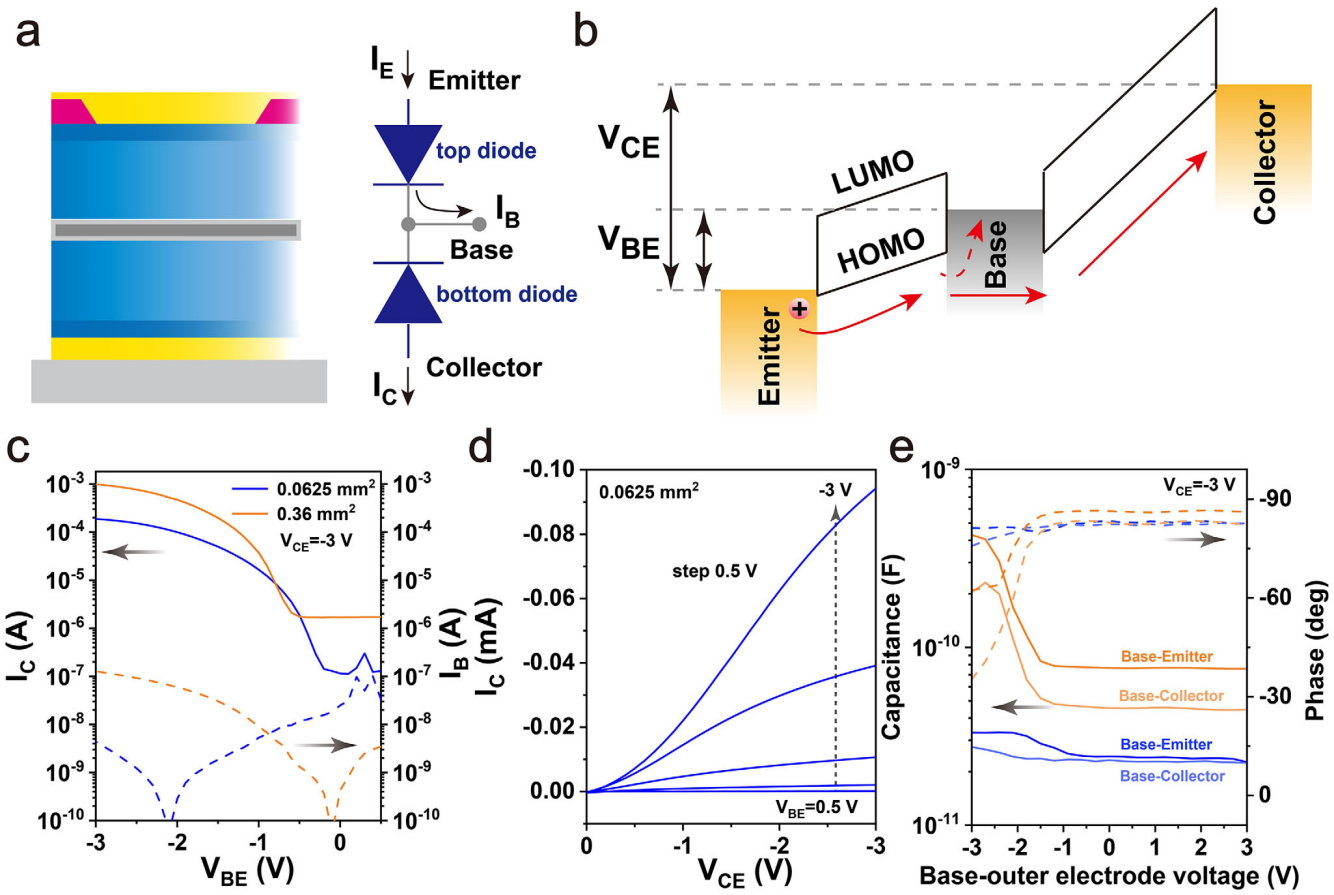
Operation mechanism of p‐type OPBTs and electrical characteristics of electrochemically anodized devices. a) The OPBTs consist of two Schottky diodes connected face to face. b) Depending on the voltage difference between the emitter and base, holes are injected at the emitter. This flow of holes is partially transmitted across the base, resulting in a transmission current *I*_*C*_, while a portion is drained by the base, producing a base leakage current *I*_*B*_. c) Transfer curves of OPBTs with an *A*_*act*_ of 0.0625 mm^2^ and 0.36 mm^2^ with a *V*_*CE*_ of ‐3 V. d) Output curves of OPBTs with an *A*_*act*_ of 0.0625 mm^2^. e) Capacitance and phase curves between base and emitter as well as base and collector of OPBTs with an *A*_*act*_ of 0.0625 mm^2^ and 0.36 mm^2^ at a constant frequency of 10 kHz. The phase in anodized samples stays close to −90°, indicating excellent insulating properties of the base oxide layer.

The working mechanism of pentacene OPBTs is illustrated in **Figure** **2**a, b. OPBTs consist of two Schottky diodes connected in a face‐to‐face configuration via the base electrode.^[^ ^42^ ^]^ The interfaces between the base and the semiconductor layers are inherently rectifying, while charge injection at the outer electrodes (emitter or collector electrode) can be approximated as Ohmic due to the insertion of doped pentacene. The diode between the emitter and base (top diode) operates in the forward direction, whereas the diode between the base and collector (bottom diode) operates in reverse (Figure 2a). The resulting alignment of the highest occupied molecular orbital (HOMO) and the lowest unoccupied molecular orbital (LUMO) energy levels is shown in Figure 2b. By controlling the emitter current through the potential difference between the base and emitter, the OPBT operates as a switch. When *V*_*BE*_ = 0 or the base is positive relative to the emitter, no holes are injected, and the transistor remains off, regardless of the collector‐emitter potential difference. However, when *V*_*BE*_ is sufficiently large and the base is negative relative to the emitter, a significant current is injected, turning the transistor on. Currents are injected at the emitter and flow toward the base, with a significant portion of the emitter current transmitted to the collector electrode. The transmission factor (*α*) quantifies this process by defining the fraction of emitter current that successfully passes through the base and reaches the collector *α* = *I*_*C*_/*I*_*E*_. A high *α* is critical for efficient operation, as it indicates minimal carrier loss in the base. Additionally, the current gain (*β*) is an essential parameter describing the amplification capability of the device, given by *β* = *I*_*C*_/*I*_*B*_. Since the base leakage current (*I*_*B*_) represents the fraction of injected carriers that do not reach the collector, minimizing *I*_*B*_ enhances the current gain. The two parameters are related as *β* = *α*/(1 − *α*). Thus, a high transmission factor directly contributes to a high current gain, making OPBTs highly suitable for applications requiring strong signal amplification. To maintain efficient charge transport and minimize unwanted charge loss at the base contact, the oxide layer must passivate the base metal while preserving pinholes that facilitate hole permeability. This ensures that a large fraction of the emitter current is transmitted to the collector, optimizing both *α* and *β* for enhanced device performance.

Then, the impact of the electrochemically anodized base on OPBTs with different *A*_*act*_ is discussed here. All electrical measurements were conducted with encapsulation (glass–glass encapsulation) at room temperature in ambient air. The SiO insulating layers have two crossed stripe‐like open windows inserted underneath the emitter electrode to reduce the *A*_*act*_ from 0.36 mm^2^ to 0.0625 mm^2^. In previous work, this insulating layer is demonstrated to improve the overall performance of n‐type OPBTs by lowering the parasitic voltage drop along the metallic contact lines and suppressing leak current emerging from outside of the area where the three electrodes overlap.^[^ ^37^ ^]^ The transfer characteristics of anodized OPBTs with varying *A*_*act*_ are shown in Figure 2c. In the transfer curves, *I*_*C*_ was measured by sweeping the *V*_*BE*_ at a constant *V*_*CE*_ of ‐3 V in a common‐emitter configuration. The performance parameters extracted from the transfer curves are summarized in **Table** **1**. The anodized base electrode‐based pentacene devices with varied areas show reliable transistor performance with low base leakage current, giving us an initial indication that the organic semiconductor material is not substantially damaged during the wet electrochemical anodization. It is noteworthy that the device with *A*_*act*_ of 0.0625 mm^2^ shows a *β*_*max*_ up to 1.89 × 10^6^ in Figure S3 (Supporting Information).

**Table 1:** The on‐current (*I*_*on*_), on‐current density (*J*_*on*_), off‐current (*I*_*off*_), on/off ratio (*I*_*on*_/*I*_*off*_), leakage current (*I*_*B*_), threshold voltage (*V*_*TH*_), maximum transconductance (*g*_*m*, *max*_), maximum transmission (*α*_*max*_), and maximum current gain (*β*_*max*_) of pentacene‐based OPBT with different *A*_*act*_.

*A*_*act*_ [mm^2^]	*I*_*on*_ [mA]	*J*_*on*_ [mAcm^−2^]	*I*_*off*_ [µA]	*I*_*on*_/*I*_*off*_	*I*_*B*_ [A] (*V*_*BE*_ = ‐3 V)	*V*_*TH*_ [V]	*g*_*m*, *max*_ [mS]	*α*_*max*_	*β*_*max*_
0.0625	0.21(±0.06)	331(±94)	0.21(±0.05)	1.03(±0.47) ×10^3^	3.10(±1.52) ×10^−8^	−1.08(±0.22)	0.18(±0.07)	99.9988(±0.0020)%	8.60(±5.17) ×10^5^
0.36	1.08(±0.37)	299(±102)	2.36(±1.06)	4.86(±1.22) ×10^2^	1.32(±0.17) ×10^−7^	−1.23(±0.07)	0.69(±0.25)	99.9975(±0.0027)%	1.80(±1.20) ×10^5^

The transistor shows improved performance over air‐oxidized devices,^[^ ^26^ ^]^ including higher *J*_*on*_, decreased *A*_*act*_, reduced *V*_*TH*_,^[^ ^44^ ^]^ lower *I*_*B*_, higher *α*_*max*_, and higher *β*_*max*_. A comparison to the previously published p‐type OPBTs is given in Table S1 (Supporting Information). It is worth noting that the *A*_*act*_ decreased by a factor of 100, enabling device miniaturization as an additional advantage. Meanwhile, the *I*_*B*_ improved by a factor of 100,000, leading to a corresponding 100,000‐fold increase in the *β*_*max*_. Furthermore, the *α*_*max*_ improved significantly, from 97% to 99.9999%. The *J*_*on*_ shows a higher value regarding the devices with an anodized base, highlighting the efficiency and effectiveness of anodization in improving OPBT functionality. Therefore, electrochemical anodization is a promising technology to be included in the OPBTs manufacturing process to achieve low leakage currents without damaging the organic semiconductor material. Figure 2d and Figure S4 (Supporting Information) show the output curves of the anodized device with *A*_*act*_ of 0.0625 and 0.36 mm^2^, respectively. The output curves show promising behavior with a high degree of current control, although both curves display slightly nonlinear *I*_*C*_‐*V*_*CE*_ characteristics, presumably caused by contact resistance. To evaluate the air stability of devices, we conducted air stability tests on encapsulated OPBTs. As shown in Figure S5 (Supporting Information), after 268 hours of air exposure, the encapsulated devices exhibited no significant degradation in electrical performance. In contrast, unencapsulated devices showed a noticeable deterioration within 4 h of exposure, indicating the necessity of encapsulation for improved air stability.^[^ ^26^ ^]^ These results confirm that the encapsulation process effectively mitigates device degradation and enhances long‐term operational reliability. To further evaluate its applicability across different material systems, we applied electrochemical anodization to rubrene‐based OPBTs. The corresponding characterization results for anodized triclinic rubrene OPBTs are shown in Figure S6 (Supporting Information). The triclinic rubrene might be a material for future developments due to its high vertical mobility.^[^ ^45^ ^]^ The fabrication details of the rubrene OPBTs can be found in the Experimental Section. The results confirm successful anodization without degradation, demonstrating the feasibility of extending this approach beyond the pentacene‐based devices. Specifically, the anodized rubrene OPBTs exhibit an *α*_*max*_ of 99.9986%, a *β*_*max*_ of 7.23 × 10^4^, and a *J*_*on*_ of 321 mAcm^−^^2^. These values are comparable to pentacene OPBTs, highlighting the versatility of electrochemical anodization in enhancing OPBT performance across different organic semiconductors.

To understand how the anodization condition affects the capacitance of the device in accumulation and depletion, we carried out an impedance spectroscopy analysis. As expected, Figure 2e demonstrates an increase in capacitance between base and emitter (*C*_*BE*_) as well as between base and collector (*C*_*BC*_) with a larger *A*_*act*_. The phase of the impedance signal at a constant frequency of 10 kHz remains close to ‐90° for smaller *A*_*act*_ devices in accumulation (negative voltage values) as well as depletion (positive voltage values), which proves the excellent blocking of base leakage current by the tight AlO_x_ layer around the base electrode formed by electrochemical anodization. The reduced phase of the impedance data for devices with a large *A*_*act*_ in the accumulation (negative voltage values) indicates less perfect oxidation, likely due to the increased probability of leakage points on the base electrode with larger areas. This observation is consistent with the device with a larger *A*_*act*_ showing a large leakage current in the transfer curve (Figure 2c). As the behavior of the capacitance is similar for the base‐emitter and base‐collector diode, we conclude that the AlO_x_ layer forms on both sides of the base electrode in a similar way, effectively passivating the entire electrode against leakage current flows. However, the bottom side of the base layer in the base‐collector diode undergoes less exposure to anodization due to passivation by the bottom pentacene layer. To investigate the conformity of the anodized film on both surfaces, we compare the capacitance of *C*_*BC*_ and *C*_*BE*_ between the bottom half diode (base and collector) and the top half diode (base and emitter), respectively. The lower capacitance *C*_*BC*_ compared to *C*_*BE*_ in both samples with varying *A*_*act*_ is likely due to variations in the oxidation film thickness beneath the base electrode. The phase of the impedance signal in the base‐collector diode also indicates a slightly lower value, suggesting imperfections in the oxidation film beneath the base electrode. This issue arises because the citric acid solution may not fully reach every part of the base‐pentacene interface during the process. Both diodes' capacitance and phase signals closely align, indicating good conformity of the oxidation layer formed on both sides through electrochemical anodization. Due to these differences in the bottom and top diode capacitance, it is hard to determine the oxide thickness from the capacitance. However, based on the specific capacitance of the base‐emitter electrode of approximately 400–600 nFcm^−2^, we can assume the oxide layer to be approximately 10–15 nm thick on both sides.

Overall, the anodized p‐type OPBTs show performance on par with the previously reported air‐oxidized devices, indicating that the anodization does not negatively affect the device performance. Moreover, the anodized devices are very robust and show a significantly higher transmission, which is due to the low base leakage current. To further substantiate our findings, we carried out a full small‐signal analysis of the devices.

**Figure 3:**
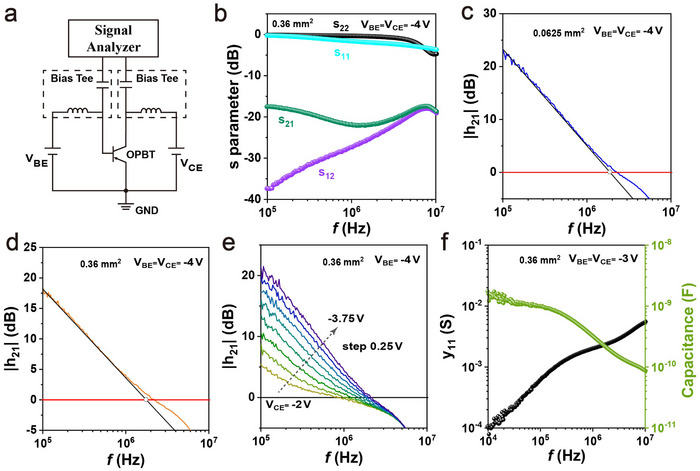
Two‐port signal analysis of OPBTs. a) Circuit diagram of the two‐port s‐parameter measurements. b) Dependence of the s‐parameters on frequency (f) for the OPBTs with the *A*_*act*_ of 0.36 mm^2^. Measured small‐signal current gain (∣*h*_21_∣) of the OPBTs with the *A*_*act*_ of 0.0625 mm^2^ c) and 0.36 mm^2^ d) plotted as a function of the measurement frequency, exhibiting a transit frequency (*f*_*T*_) of 1.89 and 1.79 MHz, respectively. The cutoff frequencies (*f*_*T*_) are determined as the frequency at which ∣*h*_21_∣ = 0 dB (red line). e) Magnitude of the small‐signal current gain (∣*h*_21_∣) of OPBTs with an *A*_*act*_ of 0.36 mm^2^ with *V*_*CE*_ ranging from ‐2 to ‐3.75 V plotted as a function of the measurement frequency. f) Base‐emitter capacitance (*C*_*BE*_) plotted as a function of the measurement frequency (f) for OPBTs with an *A*_*act*_ of 0.36 mm^2^ in the two‐port network analysis. The Base‐emitter capacitance *C*_*BE*_ was calculated from the measured admittance parameters (∣*y*_11_∣ = 2πf*C*_*BE*_).

Full two‐port s‐parameter measurements were utilized to evaluate the high‐frequency response of the pentacene OPBTs. **Figure** **3**a illustrates the circuit diagram of the characterization setup. Each OPBT was mounted on a fixture and connected using SMA cables to a signal analyzer. For applying the *V*_*BE*_ and *V*_*CE*_, two bias‐tees with the appropriate frequency response were used. A Through‐Open‐Short‐Match (TOSM) calibration was conducted before the measurement to de‐embed extrinsic effects, such as parasitic capacitances and resistances associated with the measurement setup.^[^ ^46^, ^47^, ^48^ ^]^ The two‐port s‐parameters were obtained from 100 kHz to 10 MHz, thus covering the entire range of frequencies of interest. Figure 3b illustrates a graph of the four s‐parameters plotted against frequency, which comprise a complete set of coefficients sufficient to describe the OPBT's small signal behavior at a given bias. Consequently, the hybrid parameter corresponding to the short circuit small‐signal current gain (*h*_21_ = *i*_*C*_/*i*_*B*_) was derived from the measured s‐parameters. For a more detailed description of the s‐parameter measurement setup, calibration procedure, and cutoff frequency extraction method, please refer to the Supporting Information. We note that the cutoff frequency *f*_*T*_ is defined as the frequency at which the ∣*h*_21_∣ = 0 dB.^[^ ^49^, ^50^ ^]^ The *f*_*T*_ are determined to be 1.89 MHz for the device with *A*_*act*_ of 0.0625 mm^2^ (Figure 3c) and with 1.79 MHz for 0.36 mm^2^ (Figure 3d) (both measured at *V*_*BE*_ = *V*_*CE*_ = −4 V). As illustrated in Figure 3c,d, the current gain decreases with increasing frequency, following a slope of approximately ‐20 dB per decade (indicated by the black lines in Figure 3c, d). The matching performance of small (0.0625 mm^2^) and large area devices (0.36 mm^2^) with regard to *f*_*T*_ proves that the capacitance as well as transconductance scales as expected with the area.

Since devices with different *A*_*act*_ exhibit comparable *f*_*T*_ values, we will focus on those with the *A*_*act*_ of 0.36 mm^2^ as an example in the following discussion of the *f*_*T*_ experiments. Figure 3e presents the dependence of ∣*h*_21_∣ on the OPBTs' *V*_*CE*_, where *V*_*CE*_ is varied from ‐2 to ‐3.75 V in steps of 0.25 V while keeping *V*_*BE*_ fixed at ‐4 V. As a result, *f*_*T*_ increases from 0.44 MHz at *V*_*CE*_ = ‐2 V and *V*_*BE*_ = ‐4 V, to 1.83 MHz at *V*_*CE*_ = ‐3.75 V and *V*_*BE*_ = ‐4 V. However, when *V*_*CE*_ is increased further to ‐4 V (see Figure S7, Supporting Information), *f*_*T*_ slightly decreases to 1.79 MHz. In addition, experiments were conducted by varying *V*_*BE*_ at fixed *V*_*CE*_ values of ‐3 and ‐4 V to further investigate ∣*h*_21_∣. As shown in Figure S8 (Supporting Information), for *V*_*CE*_ = ‐3 V, *f*_*T*_ increases from 0.67 MHz at *V*_*BE*_ = ‐1.75 V to a maximum value of 1.49 MHz at *V*_*BE*_ = ‐2.75 V, before decreasing to 1.31 MHz at *V*_*BE*_ = *V*_*CE*_ = ‐3 V. A similar behavior is observed in Figure S9 (Supporting Information) for *V*_*CE*_ = ‐4 V, where *f*_*T*_ increases from 0.51 MHz at *V*_*BE*_ = ‐2 V to 1.87 MHz at *V*_*BE*_ = ‐3.75 V, and then decreases to 1.79 MHz at *V*_*BE*_ = *V*_*CE*_ = ‐4 V. The dependence of *f*_*T*_ on both *V*_*BE*_ and *V*_*CE*_ is attributed to the behavior of *g*_*m*_ with respect to the applied voltages (Figure S10, Supporting Information). To evaluate the high‐frequency characteristics of the insulating properties of AlO_x_ surrounding the base electrode, which was formed by electrochemical anodization, we calculated the capacitance *C*_*BE*_ from the y‐parameters. Specifically, ∣*y*_11_∣ = 2π*fC*_*BE*_, as described by the Meyer model.^[^ ^51^ ^]^ As shown in Figure 3f, the C_*BE*_ obtained from the y‐parameters closely matches the value shown in Figure 2c. Up to a frequency of approximately 1 MHz, the capacitance value reaches the low‐frequency accumulation capacitance values as reported in Figure 2c. At higher frequencies, though, the capacitance drops significantly, also visible in the reduced slope of ∣*y*_11_∣. This behavior might be caused by an insufficient accumulation and the capacitance might ultimately reach the depletion capacitance values as shown in Figure 2e. However, this behavior might also originate from the sheet resistance limitation of the 22 nm thin base electrode.^[^ ^27^ ^]^

Overall, the small‐signal current gain analysis at high frequency is consistent with the prediction from the DC transconductance and capacitance. However, the performance of p‐type OPBTs remains below the performance of their n‐type counterparts (3–3.75 MHzV^−1^).^[^ ^23^ ^]^ The reason for this discrepancy can be mainly found in the lower transconductance. We hypothesize that this is due to either a lower vertical charge carrier mobility in the vertical direction (in‐plane π − π stacking in pentacene),^[^ ^52^ ^]^ compared to C60 used for the n‐type OPBTs, or a non‐negligible contact resistance.

### 2.3 Device Uniformity and Complementary Inverter

**Figure 4:**
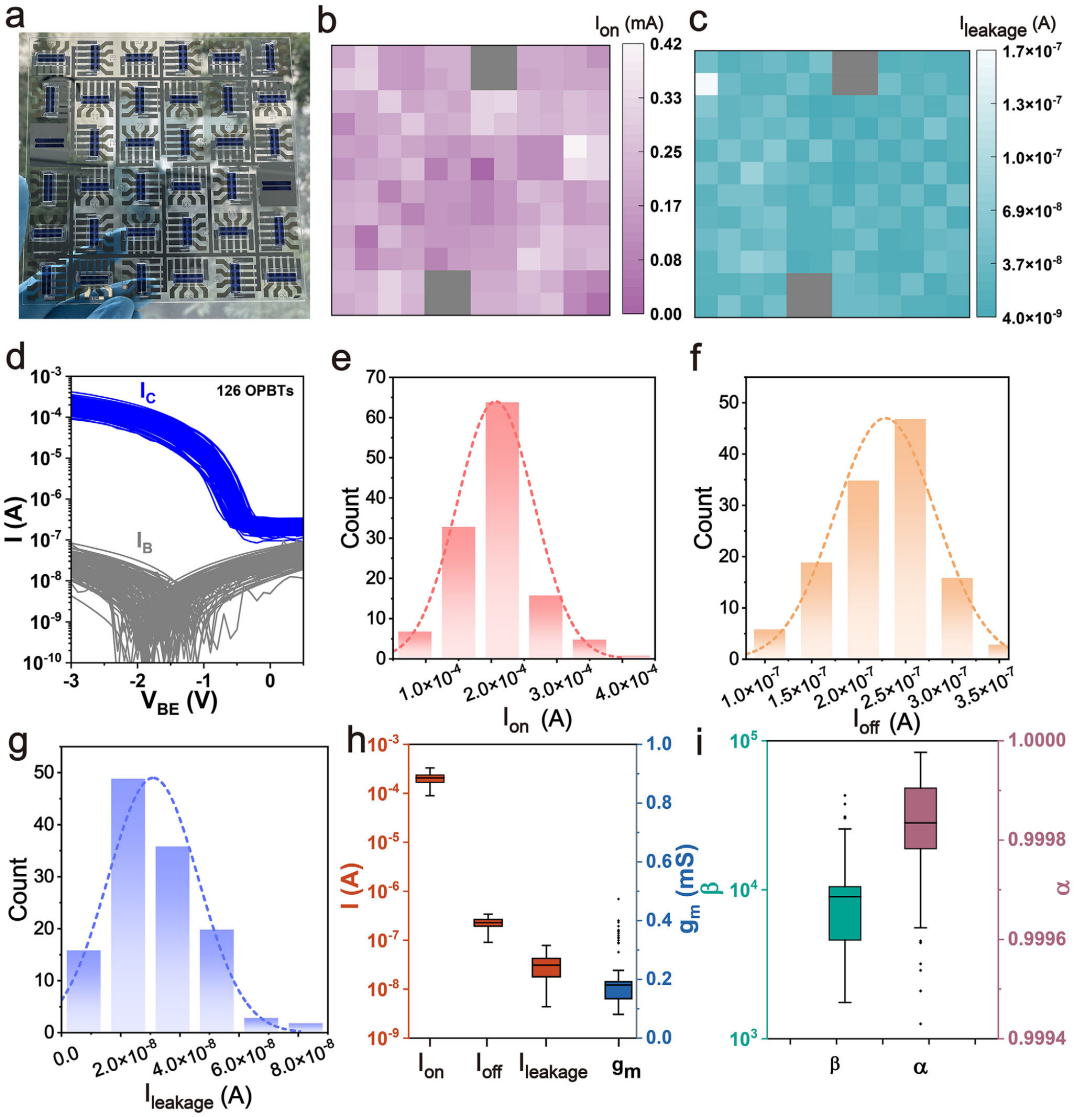
Electrical performance of a batch‐fabricated arrays of 136 p‐type OPBTs. a) Photograph of the 6 × 6 OPBT arrays (136 pixels) with electrochemically anodized base electrodes. Mapping of the *I*_*on*_ b) and *I*_*leakage*_ c) from the 136 p‐type OPBTs arrays. d) Transfer characteristic curves of the 126 working p‐type OPBTs shown in b) and c). Histogram of the *I*_*on*_ e), *I*_*off*_ f), and *I*_*leakage*_ g) extracted from the 126 transfer characteristic curves in d). The dash lines indicate the fitted normal distribution curves of *I*_*on*_, *I*_*off*_, and *I*_*leakage*_. h) Box plots of the *I*_*on*_, *I*_*off*_, *I*_*leakage*_ and transconductance *g*_*m*_ extracted from the 126 transfer characteristic curves in d). i) Current gain and transmission extracted from the 126 transfer characteristic curves in d).

To evaluate the potential for wafer‐scale fabrication and analyze the intrinsic non‐uniformity of the process, we study the performance of a large set of identical p‐type OPBT devices that can be fabricated on a single substrate simultaneously by anodization. **Figure** **4**a shows the as‐fabricated wafer‐scale pentacene OPBT arrays with the electrochemically anodized base electrode. The device‐to‐device uniformity of wafer‐scale anodized OPBTs is displayed in Figure 4, where the electrical performance of 136 OPBTs fabricated on 6 × 6 glass substrates is summarized. In Figure 4b, c, we evaluate the spatial uniformity of the OPBT arrays based on electrochemically anodized base electrode in which color maps are summarizing the *I*_*on*_ and *I*_*leakage*_ (*I*_*B*_ when *V*_*BE*_ = ‐3 V) of all 136 pixels. The grey‐colored grids in the two color maps indicate areas where no devices are present. Each grey grid covers one sample (each sample containing 4 OPBTs). These two samples are sacrificed to connect the cable and the OPBT arrays during the electrochemical anodization. As a result, 136 pixels remain in the OPBT arrays. Among these OPBTs, we only find five devices not functioning. The failure of these devices can partly be attributed to emitter‐collector shorts from particles on the sample after anodization (an effect that could be minimized in an industrial clean room environment). Therefore, the yield of electrochemical anodized OPBTs is (136 − 5)/136 ≈ 96.3%. Moreover, 5 OPBTs show larger *V*_*TH*_ but still comparable on/off ratio of the transfer curve. No obvious spatial distributions in these two color maps were detected. As shown in Figure 4c‐i, the OPBTs exhibit highly uniform electrical performance, whose average *I*_*on*_, *I*_*off*_, *I*_*leakage*_ and *g*_*m*, *max*_ are 2.1 × 10^−4^ A, 2.1 × 10^−7^ A, 3.1 × 10^−8^ A and 0.18 mS, respectively. Additionally, the maximum and minimum values can more clearly indicate the uniformity of the population. Herein, the maximum and minimum *I*_*on*_ were 4.2 × 10^−4^ A and 9.0 × 10^−5^ A, the maximum and minimum *I*_*off*_ were 3.3 × 10^−7^ A and 8.4 × 10^−8^ A, the maximum and minimum *I*_*leakage*_ were 8.3 × 10^−8^ A and 4.3 × 10^−9^ A, and the maximum and minimum *g*_*m*, *max*_ were 0.47 mS and 0.08 mS. There was no obvious relationship between these maximum or minimum values and their positions. The electrochemically anodized OPBTs exhibit exceptional performance uniformity, with an average transmission factor *α* of 99.9835% and a current gain *β* of 8982 at V_*BE*_ = ‐3 V. These values confirm that carrier transmission through the pinholes in the base electrode is highly efficient, minimizing base leakage and maximizing signal amplification. The high transmission factor *α* and current gain *β* achieved in our devices demonstrate the effectiveness of the anodization process in suppressing base leakage and optimizing charge transport. Such high uniformity and performance are crucial for the reliable operation of OPBT‐based circuits, particularly in applications requiring precise gain control and amplification.

**Figure 5:**
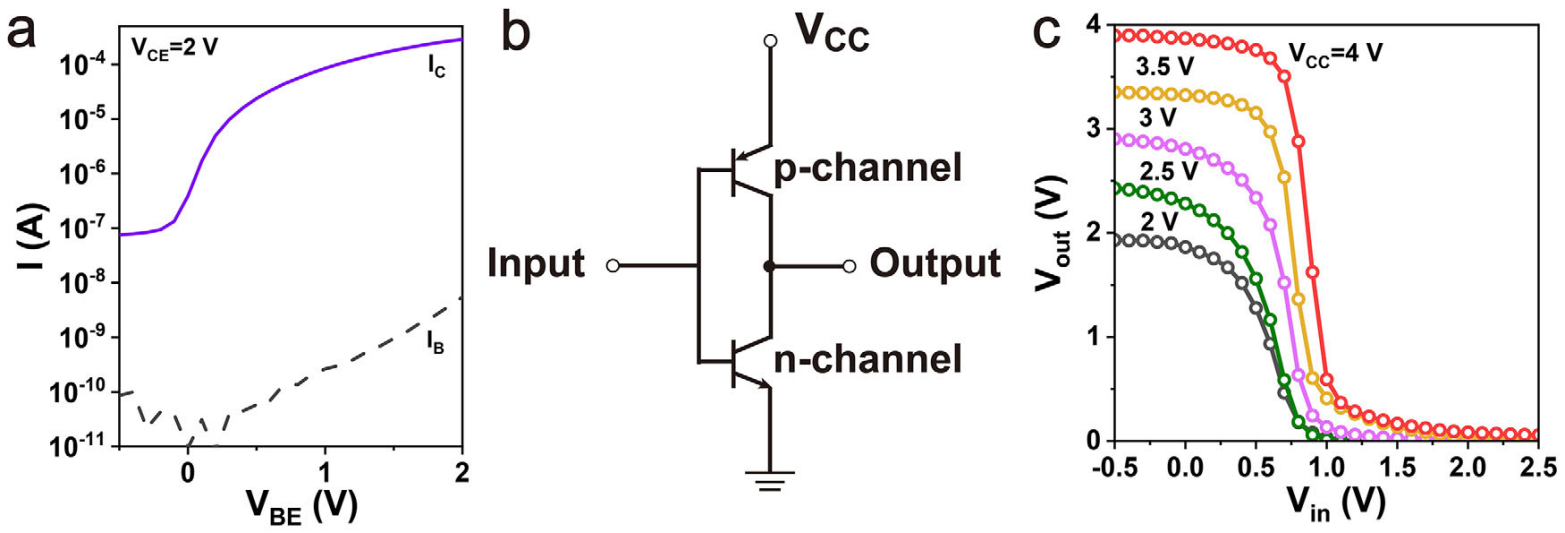
Organic complementary inverter characteristics. a) Transfer curve of C60‐based n‐type OPBTs with anodized base electrode. Circuit diagram b) and transfer curve c) of the organic complementary inverter measured at *V*_*CC*_ = 2.0, 2.5, 3, 3.5, and 4.0 V.

The excellent performance uniformity of the electrochemically anodized p‐type OPBTs, along with the large‐scale scalability of the device fabrication method, opens up the possibility for integrated circuits. To demonstrate this, we combined the electrochemically anodized p‐type and n‐type OPBT devices to construct a complementary inverter. The n‐type devices can be fabricated using the same anodization process.^[^ ^37^ ^]^ **Figure** **5**a and Figure S11 (Supporting Information) show the transfer and output curves of an n‐type OPBT based on C60 as semiconductor materials anodized at 4 V, respectively. Furthermore, Figure 5b and Figure S12 (Supporting Information) show the result of the co‐integration of the p‐ and n‐type device to form a complementary inverter. While the wafer‐scale fabrication of individual devices has been demonstrated, in this study, we focus on the electrical performance of single devices rather than large‐scale circuit integration. The complementary inverter in Figure 5b serves as a proof‐of‐concept demonstration of device functionality, laying the groundwork for future large‐scale integration. The p‐type OPBT is connected through its emitter electrode to the positive supply voltage (*V*_*CC*_) and through its collector electrode to the output terminal, where the output signal (*V*_*out*_) is measured. The n‐type OPBT, on the other hand, is connected through its collector electrode to the same output terminal and through its emitter electrode to the ground. The base electrodes of both the n‐type OPBT and the p‐type OPBT are connected together to the input signal (*V*_*in*_). The transfer curves of the inverter, recorded for various *V*_*CC*_ ranging from 2 to 4 V, are shown in Figure 5c. The transconductance and threshold voltage of both devices are not perfectly matched, however, the complementary inverter shows excellent rail‐to‐rail switching with a gain >10, which can be further optimized by matching the device's dimensions.

## 3 Conclusion

In this work, we demonstrated a novel approach for fabricating high‐performance, wafer‐scale p‐type OPBT arrays using electrochemically anodized base electrodes. This process allows for precise control of base electrode oxidation directly on top of the organic semiconductor, leading to enhanced device performance without damaging the semiconductor layer. Our anodized p‐type OPBTs achieved superior electrical characteristics, including a high *J*_*on*_ of 301 mAcm^−^^2^, low *I*_*B*_ of 4.32 × 10^−9^ A, excellent *α*_*max*_ of 99.9999%, and robust *β*_*max*_ of 1.89 × 10^6^. Our results demonstrate that electrochemical anodization offers substantial improvements in device performance, including a 100,000‐fold decrease in *I*_*B*_ and a corresponding enhancement in *β*_*max*_. The *α*_*max*_ improved from 97% to 99.9999%, establishing this method as an effective approach for advancing the capabilities of p‐type OPBTs. Moreover, our findings indicate that the anodized OPBTs can be scaled down, providing opportunities for device miniaturization and enabling the development of compact, high‐performance transistors. Impedance spectroscopy verified the formation of stable oxide films, which contribute to the reliable and consistent behavior of the devices across varying *A*_*act*_. The small signal analysis of the devices further underscores their suitability for high‐speed applications, with a *f*_*T*_ of 1.49 MHz and a voltage‐normalized transit frequency of 0.54 MHzV^−1^. Additionally, large‐scale fabrication of OPBT arrays yielded highly uniform electrical performance, with a 96.3% yield, demonstrating the viability of this technique for commercial production. Furthermore, an inverter with excellent switching performance is demonstrated by integrating anodized p‐type OPBTs with n‐type OPBTs. These results reveal the strong potential of electrochemically anodized p‐type OPBTs for integration into advanced organic electronics, including applications in active matrix displays and integrated circuits. Our method addresses key challenges in the scalability of p‐type OPBTs fabrication, paving the way for the development of complementary circuits essential for future organic electronic technologies.

## 4 Experimental Section

### Device Fabrication

Pentacene‐based OPBTs had a symmetric layer stack in the vertical direction, as shown in Figure 1a. Electrochemical anodization of the Al base electrodes was carried out after the half‐device arrays were evaporated layer by layer through a set of shadow masks on the cleaned glass substrate in an ultra‐high vacuum (10^−7^ mbar) evaporation system. The glass substrate with a size of 15 cm × 15 cm was cleaned thoroughly with N‐methylpyrrolidone, deionized water, ethanol, and an ultra‐violet ozone cleaning system. For anodization, the arrays of 34 samples with four pixels each (136 transistors in total) were grouped into two groups that share a common anodization pad. Therefore, the anodization process was carried out twice on one wafer. In the bottom half‐device, a thin chromium layer (5 nm, 0.2 Ås^−1^) improves the adhesion of the collector gold electrode (30 nm, 0.3 Ås^−1^). Next, an aluminum reinforcement layer (100 nm, 2 Ås^−1^) connecting the base electrode of all devices to common pads was evaporated, upon which a 2 wt.% F6TCNNQ doped pentacene layer (50 nm, co‐evaporation with the rate of 1 Ås^−1^ & 1 Ås^−1^), intrinsic pentacene layer (250 nm, 2 Ås^−1^) and base aluminum electrode(22 nm, 1 Ås^−1^) were evaporated. Then, the half‐fabricated OPBT arrays were taken from the vacuum chamber and annealed on the hotplate with 120 °C 2 min in the glovebox to improve the adhesion between the semiconductor layer and base electrode. Before the anodization, a nail protection polymeric coating was employed at the semiconductor layer's edge to avoid the semiconductor layer's delamination from the glass substrate. After anodization, the samples were returned to the vacuum chamber to evaporate the remaining top half‐device. Upon the base layer, the second pentacene layer (250 nm, 2 Ås^−1^) and 2 wt.% F6TCNNQ doped pentacene layer (50 nm, 1 Ås^−1^ & 1 Ås^−1^) were evaporated. A layer of SiO (100 nm, 1 Ås^−1^) was deposited to limit the contact area between the top emitter gold electrode (30 nm, 0.3 Ås^−1^) and doped pentacene layer, defining the *A*_*act*_ to 0.0625 mm^2^ (0.25 mm × 0.25 mm). For devices with a larger *A*_*act*_, the emitter electrode was directly deposited on the top of the doped pentacene layer without the SiO layer. After evaporation, the samples were transferred to the glovebox to do encapsulation with cavity glasses and epoxy glue. The CO_2_ laser was used to cut the connections among the base electrodes in the device arrays before the samples were individually cut.

For the rubrene‐based OPBTs, the bottom diode's emitter contact was in direct contact with a 75 nm‐thick p‐doped rubrene layer (1 Ås^−1^), co‐evaporated with 5 wt.% F6TCNNQ as a contact dopant. The intrinsic rubrene layer in both the bottom and top diodes has a thickness of 200 nm. In the top diode, the collector contact was in direct contact with a 70 nm‐thick p‐doped rubrene layer (1 Ås^−1^), co‐evaporated with 5 wt.% C60F48 as a contact dopant. For rubrene crystal growth, the bottom diode undergoes annealing at 130 °C for 20 min in a glovebox after depositing a 40 nm‐thick p‐doped rubrene layer, facilitating the formation of triclinic rubrene, which serves as a seed layer for the epitaxial growth of the subsequent rubrene layer. Similarly, in the top diode, annealing was performed at 150 °C for 2 min in a glovebox after depositing a 35 nm‐thick intrinsic rubrene layer, enabling the formation of triclinic rubrene as a seed layer for further epitaxial growth.

For the C60‐based OPBTs, in direct contact with the emitter‐ and collector contacts, n‐C60 20 nm (1 Ås^−1^) co‐evaporating C60 with 2 wt.% W_2_(hpp)_4_ was used as a contact dopant. Also, the thickness of intrinsic C60 is 50 and 100 nm in the bottom diode and top diode, respectively.

### Electrochemical Anodization Process

A 1 mM citric acid solution was prepared in deionized water to anodize the aluminum base electrode. The half‐fabricated OPBT arrays were taken out to ambient air and immersed into the citric acid solution, as shown in Figure S1 (Supporting Information). The common anodization pads of the aluminum base electrodes in arrays were as an anode and an aluminum sheet in solution as a counter electrode. A constant anodization potential of 4 V was applied to the base electrode by a Keithley SCS 4200 Parameter Analyzer, as shown in Figure 1b. The anodization current was applied until reaching a plateau after around 120 s to form the AlO_x_ oxidization layer around the Al base. Then, the samples were rinsed with deionized water and blown dry with *N*_2_ flow. After that, the samples were heated on a hotplate with 60 °C overnight in the glovebox.

### Device Characterization

All electrical measurements were conducted at room temperature and in ambient air. The measurement software SweepMe! (sweep‐me.net) was used for all electrical characterizations. The transfer and output characteristics of the transistors and inverter were measured with a Keithley SCS 4200 Parameter Analyzer. Impedance spectroscopy was measured with Autolab PGSTAT302N. S‐parameter measurements were performed using a Signal Analyzer Rohde & Schwarz FSV‐7 and Keysight B2912A. The SEM images were captured using a Zeiss Gemini SEM 500.

## Conflict of Interest

The authors declare no conflict of interest.

## Supporting information

Supporting Information

## Data Availability

The data that support the findings of this study are available from the corresponding author upon reasonable request.
