# Supplementary material for: Electrochemical Anodization for the Fabrication of Wafer‐Scale p‐Type Organic Permeable Base Transistors Arrays with MHz Operation
Source: Adv Mater. 2025 May 6;37(28):2419974. doi: 10.1002/adma.202419974 (PMC12272012; doi:10.1002/adma.202419974)
Supplement: Supplementary file 1 — Supporting Information [file ADMA-37-2419974-s001.pdf]

ADVANCED MATERIALS

Supporting Information

for *Adv. Mater.*, DOI 10.1002/adma.202419974

Electrochemical Anodization for the Fabrication of Wafer-Scale p-Type Organic Permeable Base Transistors Arrays with MHz Operation

Juan Wang, Amric Bonil, Jan Frede, Lautaro Petrauskas, Jörn Vahland, Tobias Antrack, Christian Matthus, Wooik Jang and Hans Kleemann**

Supporting Information

Electrochemical Anodization for the Fabrication of Wafer-Scale p-type Organic Permeable Base Transistors Arrays with MHz Operation

Juan Wang Amric Bonil Jan Frede Lautaro Petrauskas Jörn Vahland Tobias Antrack Christian Matthus Wooik Jang Hans Kleemann**

S-Parameter Measurement and Cutoff Frequency Extraction for OPBTs

The cutoff frequency (f_T) is a critical figure of merit for transistors operating in high-frequency applications. It represents the frequency at which the small-signal current gain h_{21} (also known as the short-circuit current gain) drops to unity (0 dB), meaning the output current equals the input current, and the transistor ceases to provide current gain. Mathematically, this is expressed as the frequency at which $|h_{21}| = 1$ (0 dB). In transistor characterization, h_{21} is commonly derived from the measured s-parameters (scattering parameters), which describe how electrical signals behave at the input and output of a two-port network. The relationship between h_{21} and the s-parameters is given by:

$$h_{21} = \frac{-2s_{21}}{(1 - s_{11})(1 + s_{22}) + s_{21}s_{12}}$$

where s_{21} is the forward transmission coefficient, indicating how much of the input signal is transferred to the output. s_{11} is the input reflection coefficient, measuring impedance mismatch at the input port. s_{22} is the output reflection coefficient, which quantifies how much of the signal is reflected back from the output port due to impedance mismatch. s_{12} represents the reverse transmission coefficient, describing how much of the output signal propagates back to the input. Since h_{21} is frequency-dependent, the cutoff frequency f_T is determined by measuring and plotting $|h_{21}|$ versus frequency on a logarithmic scale. The point where $|h_{21}| = 1$ (0 dB) marks the transistor's transition to frequencies where it can no longer amplify current.

The s-parameters (s_{11}, s_{12}, s_{21}, s_{22}) of our OPBTs were measured using a Signal Analyzer Rhode & Schwarz FSV-7, in conjunction with a Source Measurement Unit Keysight B2912A to apply DC bias voltages. A custom-made bias tee was used to combine the RF and DC signals, ensuring proper biasing of the device under test. Each OPBT was mounted on a measurement box with spring-loaded contacts and SMA connectors, where the cables were directly connected for signal transmission. The two-port s-parameters were measured in the frequency range of 100 kHz to 10 MHz, covering the full range of interest for or-

ganic high-frequency transistors. To ensure measurement accuracy, a Through Open Short Match (TOSM) calibration procedure was performed before testing. This is a high-precision Signal Analyzer calibration method that eliminates systematic errors associated with two-port network measurements and removes extrinsic effects such as cable losses and mismatches, bias-tee parasitics, and connector and adapter impedances. The TOSM calibration process consists of the following steps:

- Open calibration: The measurement ports are left unconnected to establish the open-circuit response.
- Short calibration: The ports are shorted to ground to measure the short-circuit response.
- Matched-load calibration: A 50 Ω reference load is used to calibrate impedance matching conditions.
- Through calibration: Both measurement cables are directly connected to each other to define the ideal transmission reference.

After calibration, the system automatically de-embeds error sources, ensuring that the recorded s-parameters accurately represent the intrinsic performance of the OPBTs.

From the measured s-parameters, we extracted the following key insights about the OPBT operation:

- Reflection Coefficients (s_{11} and s_{22})
  The input reflection coefficient (s_{11}) and output reflection coefficient (s_{22}) describe the impedance matching at the emitter and collector, respectively. In our measurements, s_{11} and s_{22} exhibit similar trends, suggesting that the emitter and collector have comparable impedance characteristics. The values of $s_{11} \approx 0$ dB and $s_{11} \approx 0$ dB indicate that most of the input power is reflected, which is expected for OPBTs acting as capacitive devices at high frequencies.
- Forward and Reverse Transmission (s_{21} and s_{12})
  The forward transmission coefficient s_{21} (signal gain) is significantly larger than the reverse transmission coefficient s_{12}, confirming the unidirectional nature of charge transport in OPBTs. The observed asymmetry ($s_{12} \neq s_{21}$) is a key transistor characteristic, indicating that the device effectively suppresses signal transmission in the reverse direction, preventing unwanted feedback.

Table S1: The operation voltage, A_{act}, on-current density(J_{on}), leakage current(I_B), threshold voltage (V_{TH}), maximum transmission (α_{max}), and maximum current gain (β_{max}) of pentacene-based OPBT with different base oxidization method.

samples	oxidization method	operation voltage	A_{act} (mm^2)	J_{on} (mA/cm^2)	I_B (A)	V_{TH} (V)	α_{max}	β_{max}
ref [26]	ambient air	-2 V	6.25	40	1.17×10^{-4}	-1.5	97%	29
this work	electrochemical anodization	-3 V	0.0625	301	4.32×10^{-9}	-1.1	99.9999%	1.89×10^{6}

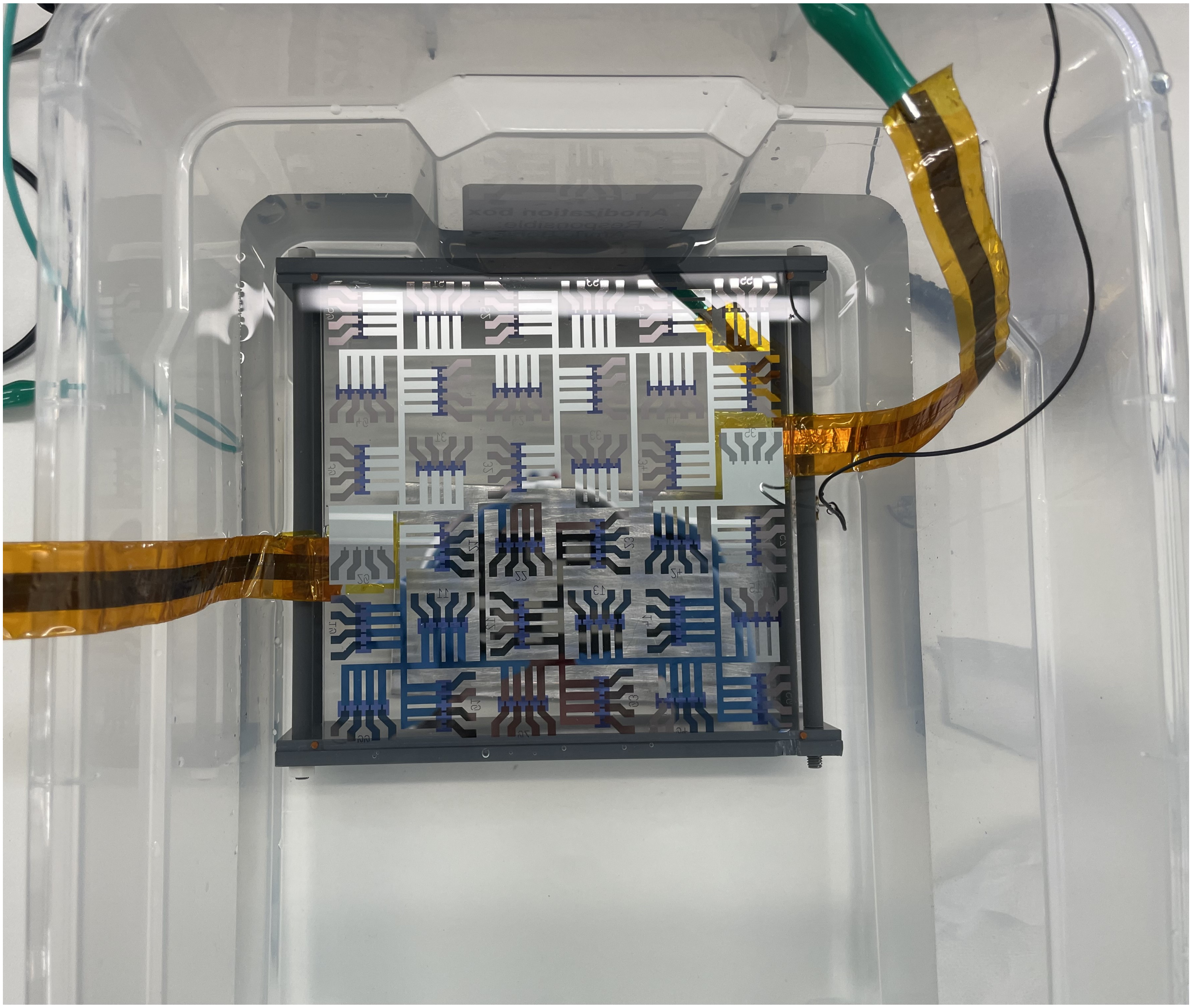

Figure S1: Wafer scale sample arrays in the anodization bath. The base electrode is contacted with a clamp and the anodization voltage is applied to the anode in the citric acid solution.

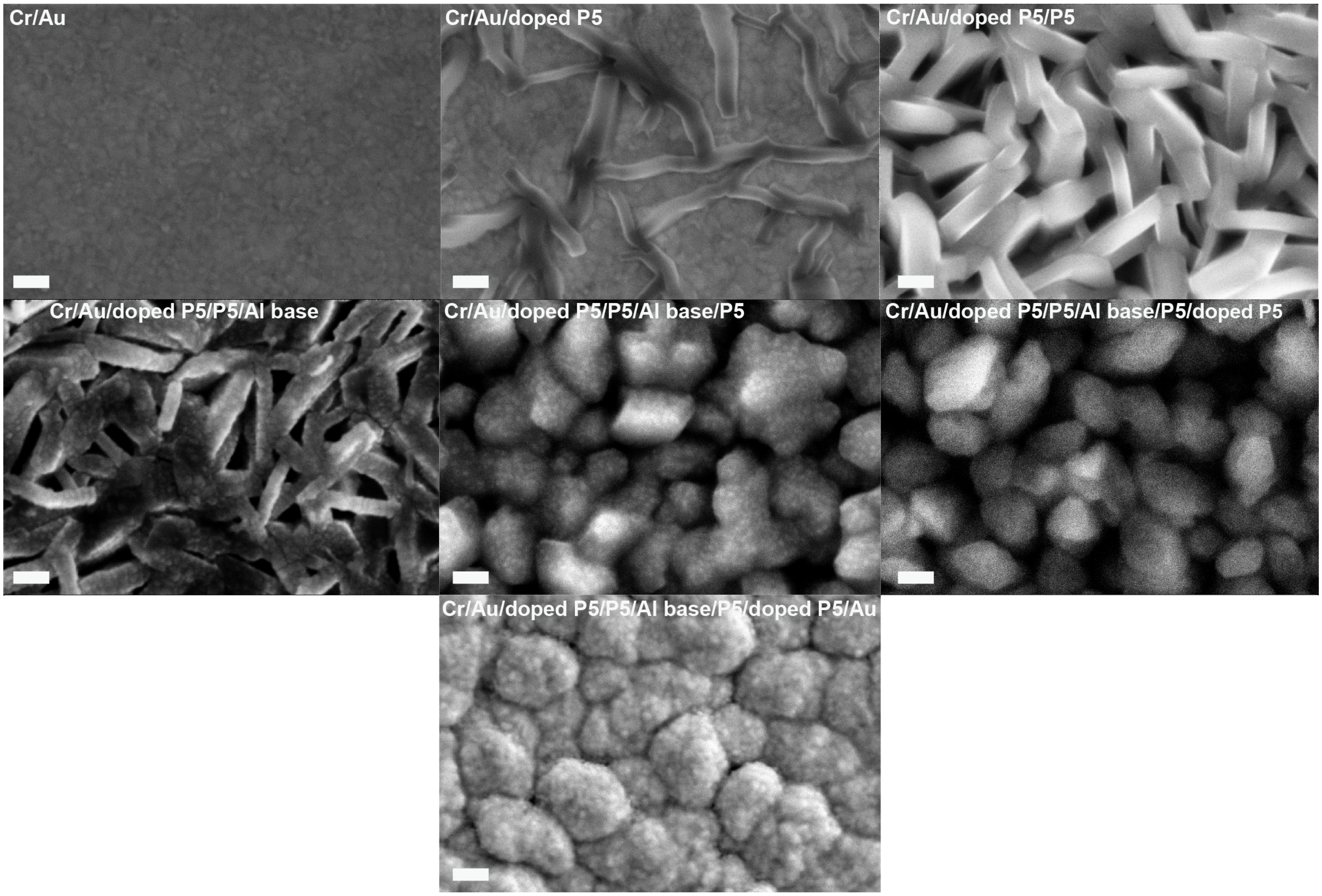

Figure S2: SEM images of each layer in anodized pentacene (P5) OPBTs. Scale bar: 100 nm.

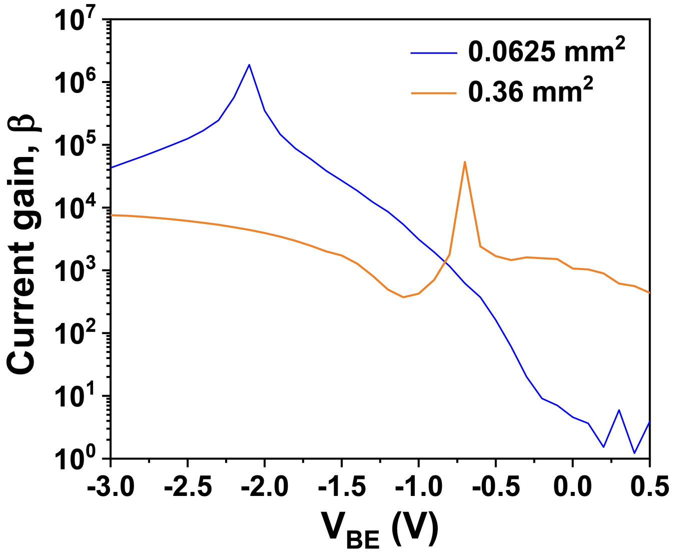

Figure S3: Current gain (β) of OPBTs with different A_{act}. The maximum current gain of 1.89×10^6 and 5.33×10^4 when A_{act} of 0.0625 mm^2 and 0.36 mm^2, respectively.

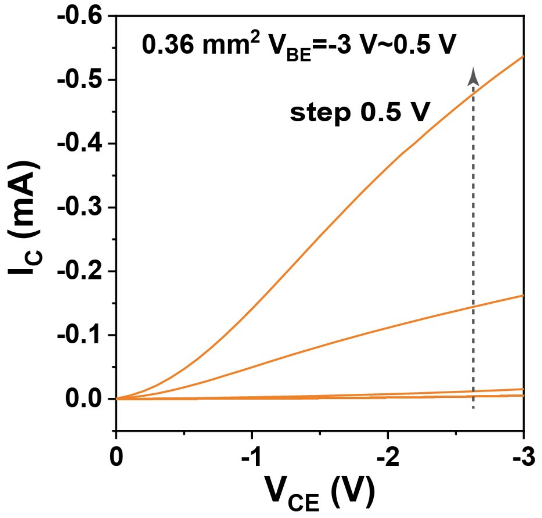

Figure S4: Output characteristics of electrochemically anodized pentacene OPBTs corresponding to the A_{act} of 0.36 mm^2.

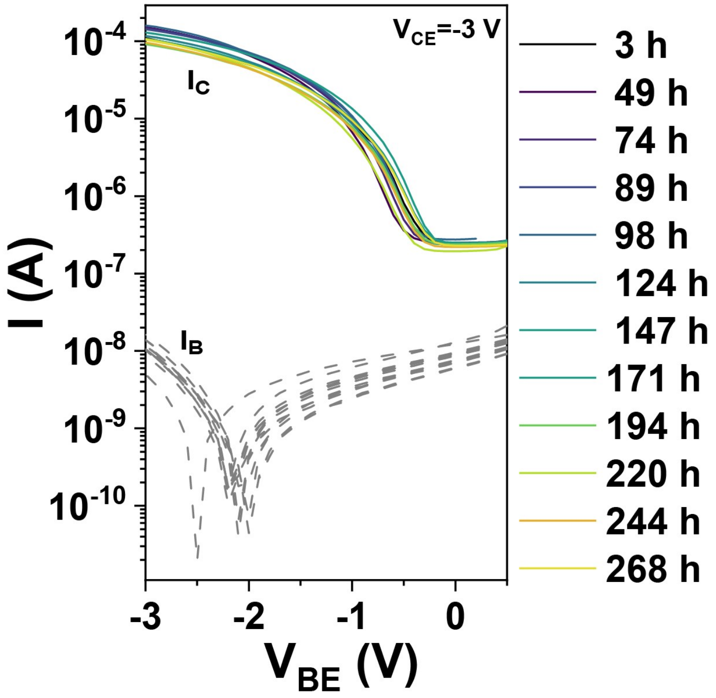

Figure S5: The evolution of the transfer curve of encapsulated devices over time.

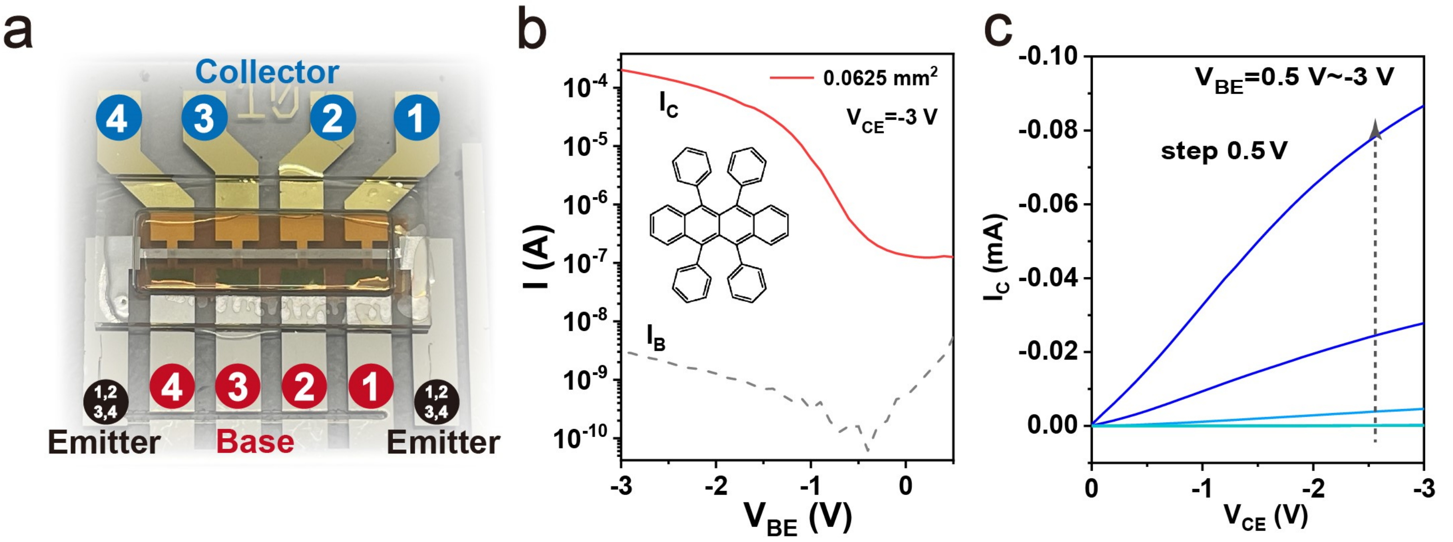

Figure S6: **a** Photograph of electrochemically anodized triclinic rubrene OPBTs on a glass substrate (edge length: 1 in.), including four active OPBT pixels. **b** Transfer curves of electrochemically anodized triclinic rubrene OPBTs with an A_{act} of 0.0625 mm^2 with a V_{CE} of -3 V. Inset: Molecular structure of rubrene. **c** Output characteristics of electrochemically anodized triclinic rubrene OPBTs.

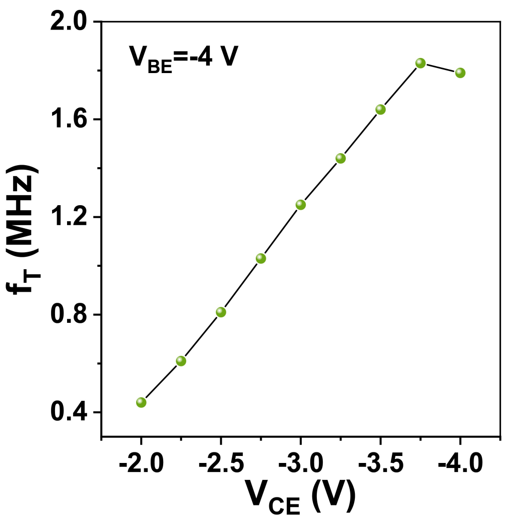

Figure S7: Cutoff frequency (f_T) plotted as a function of the collector-emitter voltage at a fixed V_{BE} of -4 V.

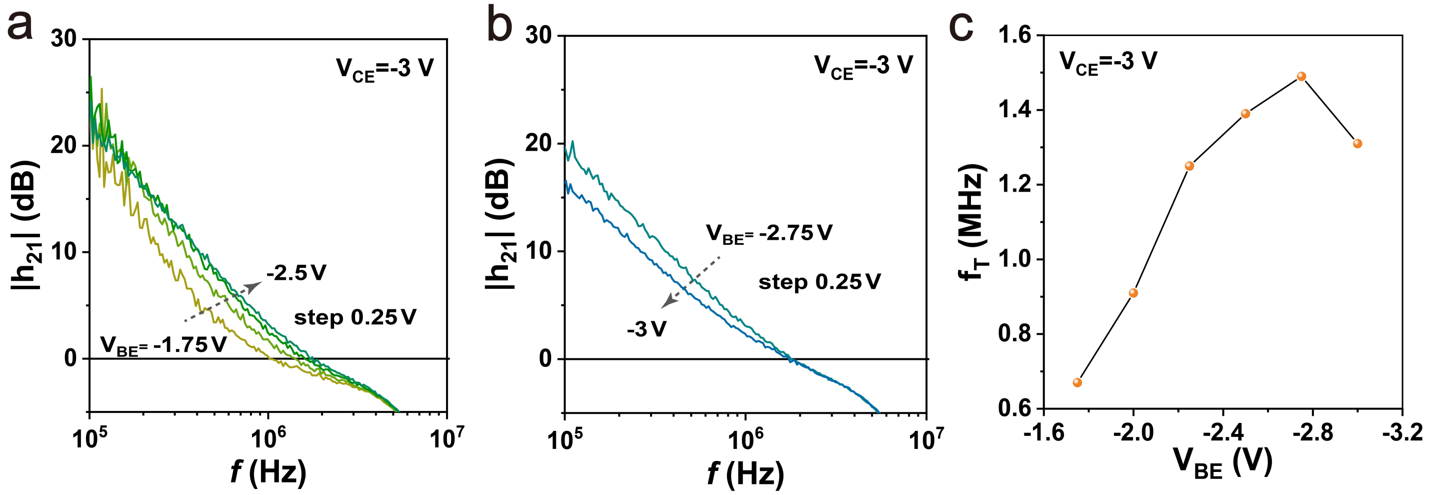

Figure S8: Magnitude of the small-signal current gain (| h_{21} |) of OPBTs with an A_{act} of 0.36 mm^2 with **a** V_{CE} ranging from -1.75 V to -2.5 V and **b** V_{CE} ranging from -2.75 V to -3.75 V plotted as a function of the measurement frequency. **c** Cutoff frequency (f_T) plotted as a function of the base-emitter voltage at a fixed V_{BE} of -3 V.

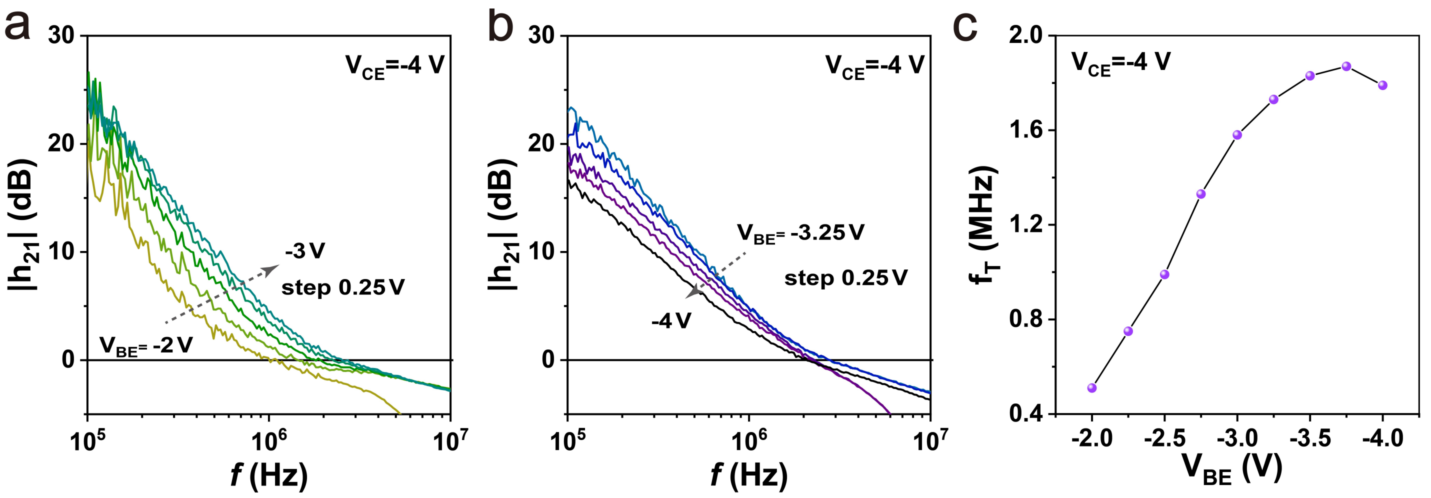

Figure S9: Magnitude of the small-signal current gain (| h_{21} |) of OPBTs with an A_{act} of 0.36 mm^2 with **a** V_{CE} ranging from -2 V to -3 V and **b** V_{CE} ranging from -3.25 V to -4 V plotted as a function of the measurement frequency. **c** Cutoff frequency (f_T) plotted as a function of the base-emitter voltage at a fixed V_{BE} of -4 V.

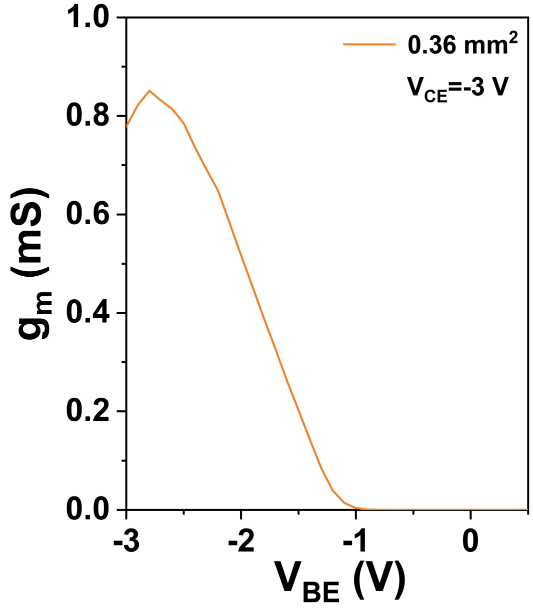

Figure S10: g_m of device with A_{act} of 0.36 mm^2 at V_{CE}=-3 V extracted from Figure 2c. The maximum g_m of 0.85 mS is obtained at V_{BE}=-2.8 V.

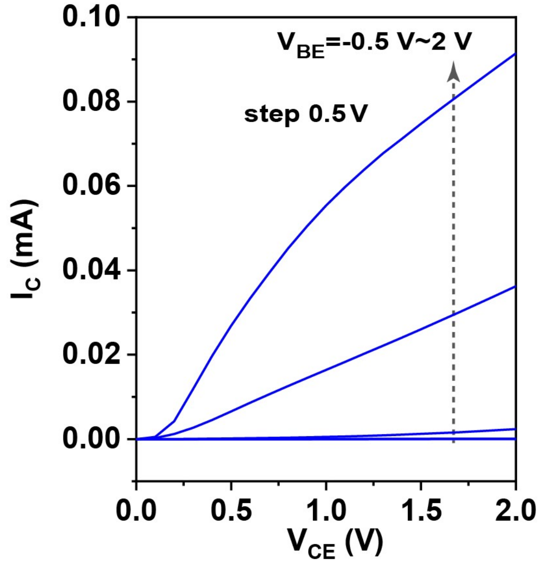

Figure S11: Output characteristics of electrochemically anodized C60 OPBTs.

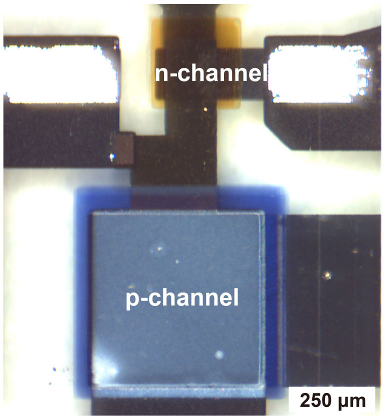

Figure S12: Photograph of an integrated organic complementary inverter.
